# Receiver Operating Characteristic Curve and Odds Ratio Should Be Used with Caution

**DOI:** 10.5812/kowsar.1735143X.735

**Published:** 2011-09-01

**Authors:** Wandong Hong, Jiansheng Wu, Xiangrong Chen

**Affiliations:** 1 Department of Gastroenterology and Hepatology,the First Affiliated Hospital of Wenzhou Medical College, Wenzhou, Zhejiang, China

**Keywords:** ROC curves, Odds ratio

Dear Editor,

We read with interest the article by Abu El Makarem MA et al. [1] entitled, “Platelet count/bipolar spleen diameter ratio for the prediction of esophageal varices: The special Egyptian situation.” This study evaluated noninvasive predictors of esophageal varices in cirrhotic patients by multivariate logistic regression and receiver operating characteristic (ROC) curve analysis. However, in our opinion, some aspects of the statistical analysis could be clarified. The first issue concerns the ROC analysis. The full area under the ROC curve (AUC) is a measure of the performance of the diagnostic test because it reflects the test performance at all possible cutoff levels. The AUC lies in the interval [0.5,1], and the larger area, the better performance. A perfect test has an AUC of 1.0, whereas random chance gives an AUC of 0.5 [2],[3]). In Abu El Makarem MA et al.’s [1] paper, the AUC for age is 0.33, which is less than 0.5. Therefore, we think that something might have gone wrong when the authors performed the ROC analysis with the SPSS software. Because both the univariate analysis and multivariate logistic regression identified age as a valuable predictor of varices, older patients experienced a higher risk having varices. We presume that the AUC for age may be 0.67 (1-0.33) rather than 0.33. The second issue has to do with the odds ratio. The odds ratio refers to a ratio of the odds of the outcome occurring in one group divided by the odds of the outcome occurring in the other group with a 1-unit increase in x [4]. The odds ratio for age was 1.205 in Abu El Makarem MA et al.’s [1] paper, but no statement such as “for a one year increase in age” was mentioned.
